# N-Acetyl-L-Leucine Accelerates Vestibular Compensation after Unilateral Labyrinthectomy by Action in the Cerebellum and Thalamus

**DOI:** 10.1371/journal.pone.0120891

**Published:** 2015-03-24

**Authors:** Lisa Günther, Roswitha Beck, Guoming Xiong, Heidrun Potschka, Klaus Jahn, Peter Bartenstein, Thomas Brandt, Mayank Dutia, Marianne Dieterich, Michael Strupp, Christian la Fougère, Andreas Zwergal

**Affiliations:** 1 German Center for Vertigo and Balance Disorders, DSGZ, Ludwig-Maximilians-University of Munich, Munich, Germany; 2 Institute of Pharmacology, Toxicology, and Pharmacy, Ludwig-Maximilians-University of Munich, Munich, Germany; 3 Department of Neurology, Ludwig-Maximilians-University of Munich, Munich, Germany; 4 Department of Nuclear Medicine, Ludwig-Maximilians-University of Munich, Munich, Germany; 5 Clinical Neuroscience, Ludwig-Maximilians-University of Munich, Munich, Germany; 6 Center for Integrative Physiology, University of Edinburgh, Edinburgh, Scotland; 7 Department of Nuclear Medicine, Eberhard Karls University of Tübingen, Tübingen, Germany; University of Otago, NEW ZEALAND

## Abstract

An acute unilateral vestibular lesion leads to a vestibular tone imbalance with nystagmus, head roll tilt and postural imbalance. These deficits gradually decrease over days to weeks due to central vestibular compensation (VC). This study investigated the effects of i.v. N-acetyl-DL-leucine, N-acetyl-L-leucine and N-acetyl-D-leucine on VC using behavioural testing and serial [^18^F]-Fluoro-desoxyglucose ([^18^F]-FDG)-μPET in a rat model of unilateral chemical labyrinthectomy (UL). Vestibular behavioural testing included measurements of nystagmus, head roll tilt and postural imbalance as well as sequential whole-brain [^18^F]-FDG-μPET was done before and on days 1,3,7 and 15 after UL. A significant reduction of postural imbalance scores was identified on day 7 in the N-acetyl-DL-leucine (p < 0.03) and the N-acetyl-L-leucine groups (p < 0.01), compared to the sham treatment group, but not in the N-acetyl-D-leucine group (comparison for applied dose of 24 mg i.v. per rat, equivalent to 60 mg/kg body weight, in each group). The course of postural compensation in the DL- and L-group was accelerated by about 6 days relative to controls. The effect of N-acetyl-L-leucine on postural compensation depended on the dose: in contrast to 60 mg/kg, doses of 15 mg/kg and 3.75 mg/kg had no significant effect. N-acetyl-L-leucine did not change the compensation of nystagmus or head roll tilt at any dose. Measurements of the regional cerebral glucose metabolism (rCGM) by means of μPET revealed that only N-acetyl-L-leucine but not N-acetyl-D-leucine caused a significant increase of rCGM in the vestibulocerebellum and a decrease in the posterolateral thalamus and subthalamic region on days 3 and 7. A similar pattern was found when comparing the effect of N-acetyl-L-leucine on rCGM in an UL-group and a sham UL-group without vestibular damage. In conclusion, N-acetyl-L-leucine improves compensation of postural symptoms after UL in a dose-dependent and specific manner, most likely by activating the vestibulocerebellum and deactivating the posterolateral thalamus.

## Introduction

Acute unilateral lesions of a peripheral vestibular organ induce characteristic ocular motor and postural deficits, e.g., spontaneous nystagmus, head roll tilt and falling to the lesion side [1, 2]. Although the vestibular deficits persist, the signs and symptoms undergo behavioral recovery over days to weeks due to central vestibular compensation (VC). VC involves multiple, synchronous and synergistic adaptations in neuronal networks of various areas of the brain [3–5].

Current concepts of VC emphasize the importance of the commissural connections between the vestibular nuclei (VN), the spinal projections to the VN, the vestibulocerebellar pathways, the multisensory thalamo-cortical networks and the stress-axis activation [4, 6–12]. To further clarify these mechanisms, a μPET-based whole brain visualization of mechanisms operating during VC was recently established in a rat model of unilateral labyrinthectomy (UL). It showed several sequential changes of the regional cerebral glucose metabolism (rCGM) over time during VC including adjustment or rCGM symmetry between the vestibular nuclei, increase of rCGM in the ipsilesional spinal trigeminal nucleus and in the vestibulocerebellum bilaterally [13].

A promising therapeutic strategy for acute unilateral vestibular disorders is the use of drugs to improve and accelerate VC. For many decades N-acetyl-DL-leucine (Tanganil) has been successfully used in clinical practice to treat acute vertigo [14, 15]. It improved postural compensation of patients who had undergone vestibular neurotomy and labyrinthectomy [16]. Similar effects were seen in a cat UL model [17]. In vitro electrophysiological recordings of VN neurons and vestibular-related networks in a guinea-pig model suggest that N-acetyl-DL-leucine may act by rebalancing the abnormal membrane potential in the VN [18]. However, the localization of its action in the central nervous system as well as its pharmacological mechanism of action have remained unclear so far.

The current study aimed to determine whether in a rat model of UL N-acetyl-D-leucine or N-acetyl-L-leucine is the pharmacologically active substance affecting extent and time course of VC. The action in the brain of the effective substance was localized by serial in vivo whole-brain [^18^F]-Fluoro-desoxyglucose ([^18^F]-FDG)-μPET before, during and after applying these agents.

## Experimental Procedures

### Animals

All animal experiments were approved by the Ethics Committee of the Ludwig-Maximilians-University of Munich and the government of Upper Bavaria and performed in accordance with the guidelines for the use of living animals in scientific studies and the EU and German Law for the protection of animals. Male Sprague-Dawley rats (mean 400 ± 20 g, age 3 months, Charles River Ltd, UK) were housed one animal per cage in a temperature- and humidity-controlled room with a 12 h light/dark cycle, with free access to food and water.

### Unilateral labyrinthectomy/ sham unilateral labyrinthectomy

Animals were anaesthetized with 1.5% isoflurane delivered up to 1.2 l/min via a mask. For surgical analgesia 1.5 mg/kg meloxicam was injected s.c. before and 3 days after surgery. After local anaesthesia with 1% lidocaine hydrochloride, a left paramedian incision was made to expose the lamboidal ridge and the external ear canal. The external ear canal was opened just anterior to the exit point of the facial nerve. With a 26-gauge needle the tympanic membrane was perforated caudally to the hammer shaft, and about 0.150 ml of a 20% bupivacaine solution was instilled into the tympanic cavity. After about 1 min the bupivacaine solution was aspirated and instilled slowly again. This was repeated three times. After the local anaesthesia was instilled, the same procedure was followed to instill 0.150 ml of a 10% solution of p-arsanilic acid, which irreversibly desensitizes the primary sensory cells of the inner ear (Vignaux et al., 2012). In the control group 0.150 ml of 0.9% saline was instilled into the tympanic cavity three times (in the following called sham unilateral labyrinthectomy). After the last thorough aspiration, the wound was closed by skin suture and for preventive antibiosis 2 mg/kg marbofloxacine was injected s.c. for 3 days.

### Exclusion criteria

Animals were excluded from the study if the following symptoms were observed:

- loss of more than 20% of the pre-treatment body weight,
- ulcer of the cornea, which could occur due to an inadvertent lesion of the facial nerve,
- bleeding from the tympanic cavity, which could prevent the diffusion of bupivacaine or p-arsanilic acid into the inner ear,
- abnormalities in behavioural scoring, e.g. convulsions, paresis or hemiataxia.

### Behavioural testing after UL

Behavioural symptoms of vestibular imbalance were scored for three components after unilateral vestibular ablation [19]: nystagmus, head roll tilt and postural asymmetry. Each component was given a maximum score of 10:

- *Nystagmus* was observed visually. Intensity of spontaneous nystagmus was scored with 6–10 points, with 1 point for every 60 beats per minute (bpm). If spontaneous nystagmus was absent at rest, the animal was touched slightly. If this evoked nystagmus, a score of 1–5 points was given, with 1 point for every 60 bpm.
- Spontaneous *head roll tilt* was scored by estimating the angle between the jaw plane and the horizontal; 10 points were given either for a 90-degree (deg) angle or if the animal rested recumbent on the lesion side or showed barrel-rolling towards that side. Seven points correlated with a 60-deg and 5 points with a 45-deg angle.
- *Postural deficits* were scored as follows: spontaneous barrel rolling—10 points; barrel rolling evoked by a light touch or air-puff—9 points; recumbent position on lesion side without leg support—8 points; some ipsilesional leg support—7 points; moving around on one side or using ipsilesional legs for recumbent support—6 points; moving around with bilateral leg support—5 points; moving around with occasional falls to the ipsilesional side—4 points; moving around leaning towards the ipsilesional side—3 points; hardly noticeable asymmetry—2 points; postural asymmetry only noticeable when picked up—1 point;

Behavioral testing was done by two experienced investigators who were blinded for the treatment applied to the rats.

### Statistics

Statistical group analysis for behavioural scoring was performed with IBM SPSS and SAS Statistics software. To test for overall differences between groups a general multi-factor ANOVA (mixed effect model of variance analysis with repetition (Bonferroni-corrected)) was applied (p<0.05). To compare groups at specific time points a non-parametric t-test as used. A significance level of p<0.05 was set for rejection of the null hypothesis.

### μPET imaging

**Fig 1:**
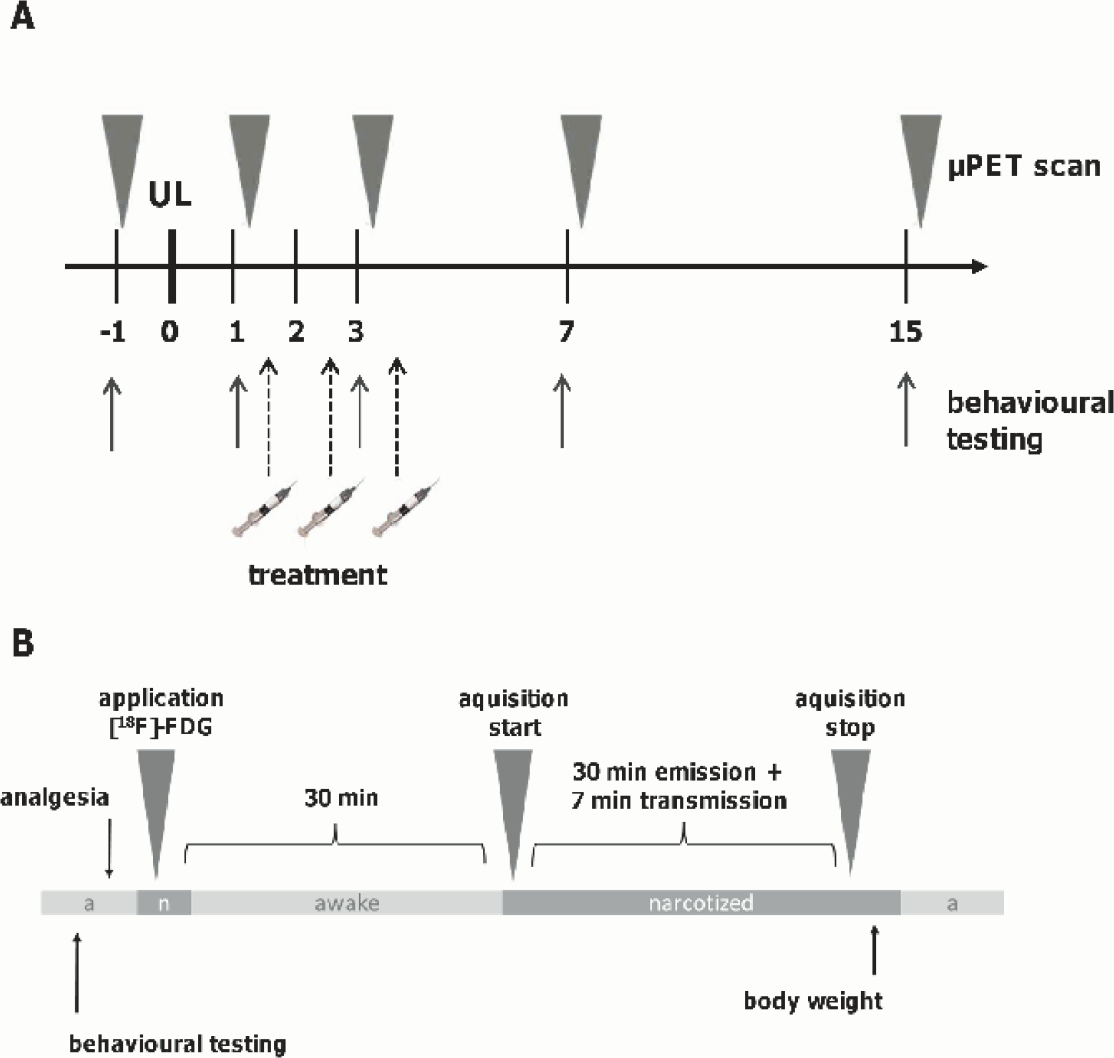
Study design and time course of treatment, μPET scanning and behavioural testing. A) N-acetyl-DL-leucine, N-acetyl-L-leucine or N-acetyl-D-leucine was administered intravenously on days 1, 2 and 3 after unilateral labyrinthectomy (UL). Sequential whole-brain [^18^F]-FDG-μPET and behavioural testing was done before and on days 1, 3, 7, 15 after UL. B) At the respective time points after behavioural testing, anaesthesia was induced with isoflurane and [^18^F]-FDG was injected. After awaking, animals were allowed to move freely until anaesthesia was induced again with isoflurane for the μPET-scan. The scan was started 30 min after [^18^F]-FDG injection. A 30-min emission recording was initiated followed by a 7-min transmission scan.

Anaesthesia was induced with isoflurane (as described above), and a cannula was placed in a tail vein. Before awaking from the anaesthesia, the animals were injected with a [^18^F]-FDG (50 MBq) bolus (in 0.5 ml saline) and were allowed to move freely until anaesthesia was induced again with isoflurane (2.5%) for the μPET-scan. The scan was started 30 min after the [^18^F]-FDG injection. Animals were positioned in the Siemens Inveon P120 PET scanner (Siemens Medical Solutions, Germany) and were kept warm with a heating pad. To prevent head movements, the head position was fixed using a custom-made head holder. A 30-min emission recording was initiated followed by a 7-min transmission scan using a rotating [^57^Co] point source. After anaesthesia, the rats were returned to their home cages (Fig. 1).

### Image processing & statistical analysis

Emission recordings were reconstructed with iterative reconstruction employing the Ordered Subsets Expectation Maximization (OSEM-3D) algorithm, which includes scatter and attenuation correction (Siemens Medical Solutions, Germany) and results in a final 128 × 128 × 159 matrix. For attenuation correction, the corresponding transmission measurements at the end of the emission scan were used. The voxel dimensions of the reconstructed images were 0.59 × 0.59 × 0.79 mm^3^. The regional cerebral glucose metabolism (rCGM) image was defined as the brain image divided by the value of global-mean-activity of the brain. Further data processing and statistical analysis were performed by means of custom-made toolboxes, implemented in statistical parametric mapping software SPM5 (Wellcome Department of Cognitive Neurology, London). All individual [^18^F]-FDG-μPET images were stereotactically normalized to a custom-made [^18^F]-FDG template (average of 20 healthy rats), which was manually coregistered to a digital high-resolution cryosection-based atlas of the rat brain, using automated algorithms implemented in SPM5. All scans were analysed after normalization to the whole brain. The normalization prior to voxel-based statistics was done with a whole brain anatomic mask, which was used for all subjects in order to eleminate the effects of the differences in the overall count. Images were compared in a voxel-wise manner for an intergroup analysis.

### Solubilisation process of N-acetyl-leucine (preparation of 30 ml)

Solubilisation of N-acetyl-DL-leucine, N-acetyl-D-leucine and N-acetyl-L-leucine were solubilized as follows (dosage 24 mg in 1 ml NaCl, based on 60 mg/kg in 2.5 ml NaCl): 6.9 ml of an aqueous solution of NaOH 20% w/v was added to 15 ml of 0.9% NaCl solution in a polypropylene flask (pH of the mixture about 13.4). Then 750 mg of the respective substance was added and afterwards the pH was adjusted to 7.5 ± 0.5 with HCl 10N and NaOH 10% w/v solutions, which were prepared in water for injection. The volume was finally adjusted to 30 ml with 0.9% NaCl solution. For the dose-response study of N-acetyl-L-leucine a stock solution was prepared at a concentration suitable for administrating 60 mg/kg. This stock was diluted each day, adjusted to body weight, for the administration of the different doses (3.75, 15, 60 mg/kg). In order to countercheck for the effective doses administered, N-acetyl-L-leucine concentrations in the prepared solution samples were analyzed. For intravenous purposes the vehicle was filtered under air flow through a 0.22μm filter (Millex GV/Durapore ref SLGV 033RS). The vehicle consisted of an aqueous solution of NaOH 20% w/v and 0.9% NaCl w/v in water for injection, at pH 7±0.5.

### Study design

The study was carried out in four steps:

1. To test for the differential treatment effects 24 rats underwent chemical UL and were divided randomly into four groups to be treated with N-acetyl-DL-leucine (DL-group), N-acetyl-L-leucine (L-group), or N-acetyl-D-leucine (D-group) at a total dosage of 24 mg i.v. per rat, equivalent to 60 mg/kg body weight, or vehicle (control group). Treatment was administered once daily on days 1, 2 and 3 post UL. Therefore, a catheter was applied under isoflurane anaesthesia in one of the lateral tail veins for each treatment injection and 1 ml of saline solution or 24 mg of the substrate in a volume of 1 ml NaCl was injected slowly within 10 sec. Subsequent behavioural scoring was performed on days 1, 3, 7 and 15 post UL in all rats. On days 1 and 3 behavioural scoring was performed prior to treatment to avoid the disruptive effect of anaesthesia.
2. On the basis of the outcome of the experiment described above, another 26 rats underwent an identical procedure to test for the dose-response effect of N-acetyl-L-leucine. On days 1, 2 and 3 N-acetyl-L-leucine was administered i.v. once a day, in a dosage of 3.75, 15 and 60 mg/kg body weight in 1 ml NaCl or vehicle, adjusted to the individual body weights.
3. To determine the effects of N-acetyl-L-leucine or N-acetyl-D-leucine treatment on neuronal plasticity during VC another 18 rats underwent UL and were divided randomly into three groups, to be treated with L-enantiomer, D-enantiomer or vehicle solution. [^18^F]-FDG-μPET recordings were made at baseline, on days 1, 3, 7 and 15 post UL in all rats. Treatment was administered once a day, on days 1, 2, and 3 post UL in a dosage of 24 mg i.v. per rat. Considering a putative influence of anaesthesia, behavioural scoring was completed before μPET scanning.
4. To test for specificity of the N-acetyl-L-leucine effect on VC another 6 rats underwent sham UL (without inner ear damage) and were treated with N-acetyl-L-leucine on days 1, 2 and 3 post sham UL (24 mg i.v. per rat). [^18^F]-FDG-μPET recordings were made at baseline, on days 1, 3, 7 and 15 post sham UL in all rats. Images were compared to the UL-group with N-acetyl-L-leucine treatment (24 mg i.v. per rat) at the respective time points.

## Results

### Effects of N-acetyl-DL-leucine, N-acetyl-D-leucine and N-acetyl-L-leucine on behavioural compensation after chemical UL

After chemical UL all animals exhibited severe signs of vestibular imbalance including nystagmus, head roll tilt and postural instability. *Nystagmus* disappeared within the first 3 days post UL. *Head roll tilt* persisted until day 15. There were no differences in extent of nystagmus or head roll tilt between the UL-groups treated with N-acetyl-DL-leucine (DL-group), N-acetyl-L-leucine (L-group) or N-acetyl-D-leucine (D-group) and the sham-treated group (SH-group) at any time point. After sham UL no animal showed signs of a vestibular damage (data not shown).

A general ANOVA showed significant differences in *postural imbalance* during the recovery process for the DL-group (p<0.04) and the L-group (p<0.0006), but not for the D-group (p = 0.75), as compared to the SH-group. The DL-group (p<0.03) and the L-group (p<0.0015) were also significantly different from the D-group.

**Fig 2:**
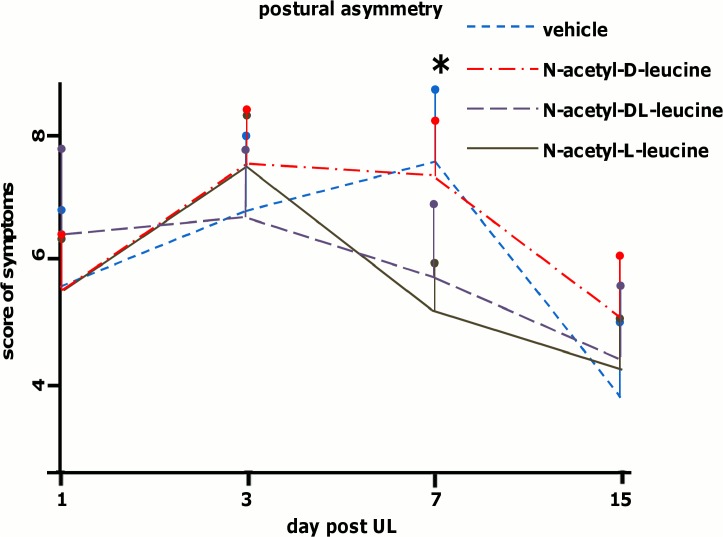
Postural imbalance scores after unilateral labyrinthectomy in the control and treatment groups. Sham treatment (vehicle), N-acetyl-DL-leucine, N-acetyl-L-leucine or N-acetyl-D-leucine (24 mg per rat each) was administered i.v. on days 1, 2 and 3 (six animals in each group). Postural imbalance scores were analyzed in all groups on days 1, 3, 7 and 15. Postural imbalance scores were significantly decreased in the N-acetyl-DL-leucine- (p < 0.03) and N-acetyl-L-leucine-group (p < 0.01) on day 7. Values are depicted as mean, the standard deviation as error bars. Significant values (DL-group vs. control and L-group vs. vehicle group on day 7) are marked with an asterisk.

Postural imbalance increased in all groups within the first 3 days due to delayed oto-toxic effects of p-arsanilic acid. No significant differences in the peak score of postural imbalance between the groups (SH-group: 7.8 ± 2.7, DL-group: 6.5 ± 1.2, L-group: 7.8 ± 0.5, D-group: 6.7 ± 1.1) were observed (Fig. 2). In the SH-group, the postural imbalance score decreased significantly until day 15 (3.6 ± 1.5). Postural imbalance scores showed a relative reduction of 53.8% on day 15 compared to the peak of symptoms. Animals treated with N-acetyl-DL-leucine (24 mg i.v. per rat, equivalent to 60 mg/kg body weight) already showed a reduction of symptoms on day 7 (5.3 ± 0.8), which further decreased to a score of 3.7 ± 0.6 on day 15. The overall relative reduction on day 15 compared to the peak of symptoms was 43.0%. Compared to the SH-group, the reduction of postural symptoms in the DL-group was significantly greater on day 7 (t-test: p < 0.03). The course of postural compensation in the DL-group was displaced forward by about 6 days relative to controls.

In the group treated with N-acetyl-L-leucine (24 mg i.v. per rat, equivalent to 60 mg/kg body weight), the postural imbalance scores decreased beginning on day 7 (4.8 ± 0.5, relative reduction compared to day 3: 38.4%) and continuing until day 15 (3.0 ± 0.7, relative reduction compared to day 3: 61.5%). Compared to the scores of the SH-group, postural imbalance scores in the L-group were significantly different on day 7 (t-test: p < 0.01). N-acetyl-L-leucine treatment accelerated the course of postural compensation by about 6 days. In the group treated with N-acetyl-D-leucine (24 mg i.v. per rat, equivalent to 60 mg/kg body weight), the postural imbalance score decreased between days 7 and 15 (3.0 ± 0.3, relative reduction compared to day 3: 55.2%), but it was not significantly different from that of controls at any time point tested.

### Dose-dependent effects of N-acetyl-L-leucine on postural compensation after chemical UL

**Fig 3:**
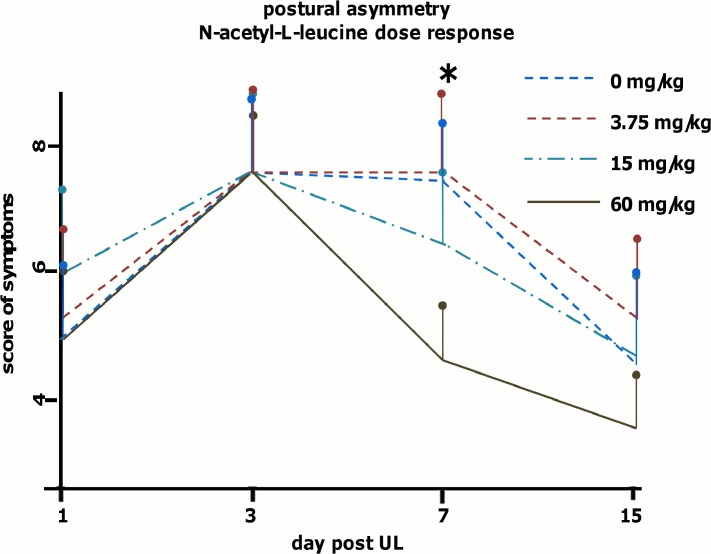
Dose-dependent effects of N-acetyl-L-leucine on postural compensation after unilateral labyrinthectomy. Sham treatment (vehicle) and N-acetyl-L-leucine (3.75, 15 or 60 mg/kg) was administered i.v. on days 1, 2 and 3 (six animals per group). Postural imbalance scores were analyzed in all groups on days 1, 3, 7 and 15. Postural imbalance was significantly decreased in the 60 mg/kg (p<0.004) but not in the 15 mg/kg or 3.75 mg/kg group. Values are depicted as mean, the standard deviation as error bars. Significant values (60 mg/kg vs. vehicle and 60 mg/kg vs. 3.75mg at day 7) are marked with an asterisk.

To check for dose-response effects of N-acetyl-L-leucine another four groups of rats (6 animals per group) were tested for postural imbalance (on days 1, 3, 7 and 15) after administration of 3.75, 15, 60 mg/kg or vehicle on days 1, 2 and 3 after UL. The administered dosages of N-acetyl-L-leucine (on days 1, 2 and 3) were analysed to assess accuracy of preparation. A general ANOVA indicated a significant difference only for the 60 mg/kg group as compared to controls (p<0.0001) and the 3.75 mg/kg group (p<0.0002). As described before, all animals showed severe symptoms of vestibular imbalance due to UL. The symptoms of postural asymmetry peaked on day 3; there were no significant differences of peak scores between groups (vehicle: 8.5 ± 0.8, 3.75 mg/kg group: 7.5 ± 0.6, 15 mg/kg group: 7.6 ± 0.6, 60 mg/kg group: 7.3 ± 1.4). The vehicle group showed a 21.2% improvement of postural imbalance scores from day 3 to 7 and 37.6% from day 3 to 15; the 60 mg/kg group showed a 41.0% reduction from day 3 to 7 and 42.4% from day 3 to 15; the 15 mg/kg group showed a reduction of 15.7% from day 3 to 7 and 38.2% from day 3 to 15; the 3.75 mg/kg group showed a reduction of 26.7% from day 3 to 15. Data analysis revealed a significant difference of postural imbalance on day 7 only for the 60 mg/kg group as compared to the vehicle group (t-test: p < 0.004), but not for the 15 mg/kg and 3.75 mg/kg group (Fig. 3).

### Effects of N-acetyl-L-leucine and N-acetyl-D-leucine on cerebral plasticity during vestibular compensation

In the current study, all UL-groups and to a lesser extent the sham UL-group showed a significant decrease of rCGM in the contralesional inferior colliculus and the auditory cortex at all time points when compared to the baseline condition before UL/ sham UL (see S1 and S2 Dataset).

**Fig 4:**
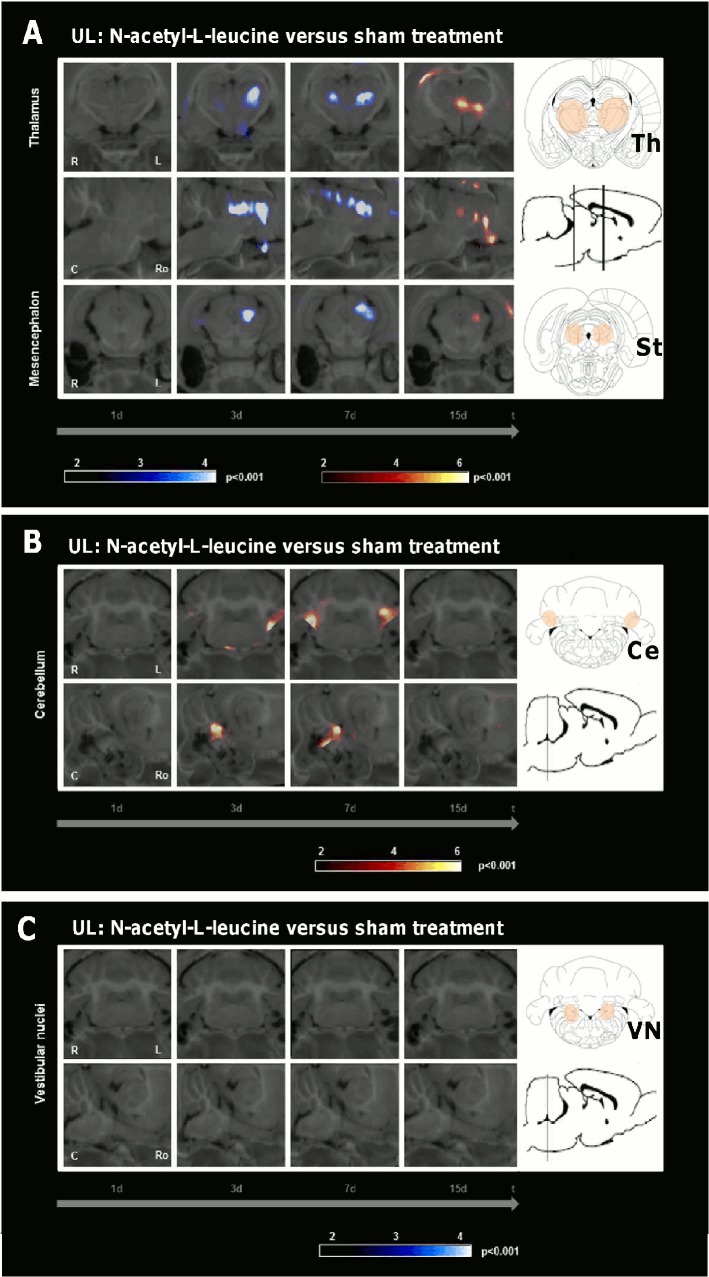
Regional cerebral glucose metabolism after N-acetyl-L-leucine as compared to sham treatment following unilateral labyrinthectomy. The images depict group-wise comparisons of the regional cerebral glucose metabolism (rCGM) on days 1, 3, 7 and 15 after unilateral labyrinthectomy (UL). A) Compared to the sham treatment group, the N-acetyl-L-leucine group (24 mg i.v. per rat) showed on day 3 a significant decrease of rCGM in the ipsilesional, on day 7 in the ipsilesional>contralesional posterolateral thalamus and subthalamic region. On day 15 an increase of rCGM was found in the centromedian thalamus and subthalamic region. B) On day 3 rCGM was significantly increased in the ipsilesional, on day 7 in the bilateral paraflocculus/flocculus (vestibulocerebellum) in the N-acetyl-L-leucine group. C) There was no decrease of rCGM in the vestibular nuclei. R right, L left, C caudal, Ro rostral, Ce cerebellum, St subthalamic region, Th thalamus; p-value < 0.001

Comparison of the UL-group treated with N-acetyl-L-leucine (24 mg i.v. per rat) and the sham-treated UL-group (0.9% saline) revealed the following rCGM changes (Fig. 4, Table 1): First, on day 3 there was a significant decrease of rCGM in the ipsilesional, on day 7 in the ipsilesional>contralesional posterolateral thalamus and subthalamic region in the L-group. On day 15 anincrease of rCGM was found in the centromedian thalamus and subthalamic region. Second, on day 3 rCGM was significantly increased in the ipsilesional, on day 7 in the bilateral paraflocculus/flocculus (vestibulocerebellum) in the L-group. Third, there was no difference of rCGM in the vestibular nuclei or any other cerebral region at any time point.

**Table 1:** shows all significant regional cerebral glucose metabolism (rCGM) changes compared between the different experimental groups as a function of time.

	**UL: N-acetyl-L-leucine versus**				**UL: N-acetyl-L-leucine versus**			
	**sham treatment**				**N-acetyl-D-leucine**			
	1 day	3 day	7 day	15 day	1 day	3 day	7 day	15 day
Thalamus		*8,92 (L)*	*9,05 (L)*	***6,37 (L)***		*6,7 (R)*	*8,72 (R)*	
			*4,31 (R)*	***8,43 (R)***			*7,48 (L)*	
Mesencephalon			*6,51 (L)*	***4,78 (L)***		*6,26 (L)*		
Cerebellum		**5,87 (L)**	**4,61 (L)**			**7,44 (L)**	**7,05 (R)**	
			**6,80 (R)**				**6,53 (L)**	
	**UL versus sham UL:**				**UL: N-acetyl-D-leucine versus**			
	**N-acetyl-L-leucine**				**sham treatment**			
	1 day	3 day	7 day	15 day	1 day	3 day	7 day	15 day
Thalamus		*6,39 (R)*	*5,84 (R)*				**5,56 (L)**	
		*7,34 (L)*	*5,25 (L)*					
Mesencephalon			*7,43 (R)*					
Cerebellum		**7,28 (R)**	**6,68 (R)**					
		**6,09 (L)**	**11,36 (L)**					

Top left: comparison of N-acetyl-L-leucine and sham treatment following unilateral labyrinthectomy (UL); top right: comparison of N-acetyl-L-leucine and N-acetyl-D-leucine treatment following UL; bottom left: comparison of N-acetyl-L-leucine treatment following UL and N-acetyl-L-leucine treatment following sham UL; bottom right: comparison of N-acetyl-D-leucine and sham treatment following UL. Decrease of rCGM is depicted in Italic, increase in Bold. The significance of the clusters is depicted by t-values at a p-value of 0.001. Degrees of freedom: (n_1_–1)+(n_2_–1) L left side, R right side

**Fig 5:**
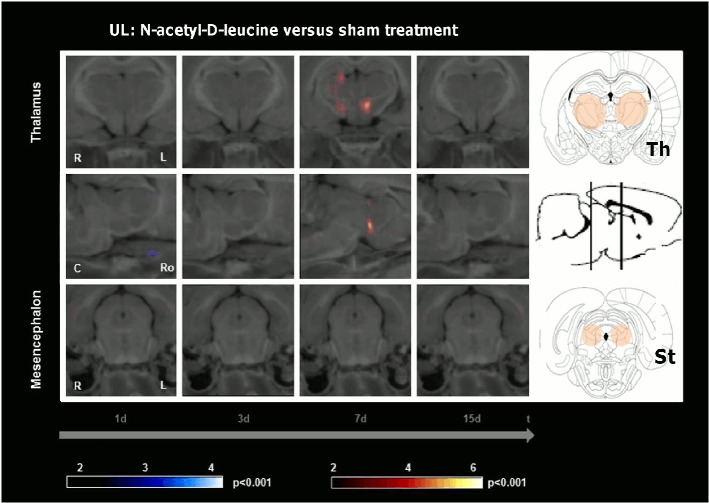
Regional cerebral glucose metabolism after N-acetyl-D-leucine as compared to sham treatment following unilateral labyrinthectomy. The images depict group-wise comparisons of the regional cerebral glucose metabolism (rCGM) on days 1, 3, 7 and 15 after unilateral labyrinthectomy (UL). The N-acetyl-D-leucine group (24 mg i.v. per rat) showed an increase of rCGM in the centromedian thalamus and hypothalamic area compared to the sham treatment group on day 7. There were no further differences of rCGM in any other cerebral region at any other time point. R right, L left, C caudal, Ro rostral, St subthalamic region, Th thalamus; p-value < 0.001

An increase of rCGM in the centromedian thalamus and hypothalamic area was found on day 7 when comparing the UL-group treated with N-acetyl-D-leucine (24 mg i.v. per rat) to the sham-treated UL-group (Fig. 5, Table 1). There were no further differences of rCGM in any other cerebral region at any other time point.

**Fig 6:**
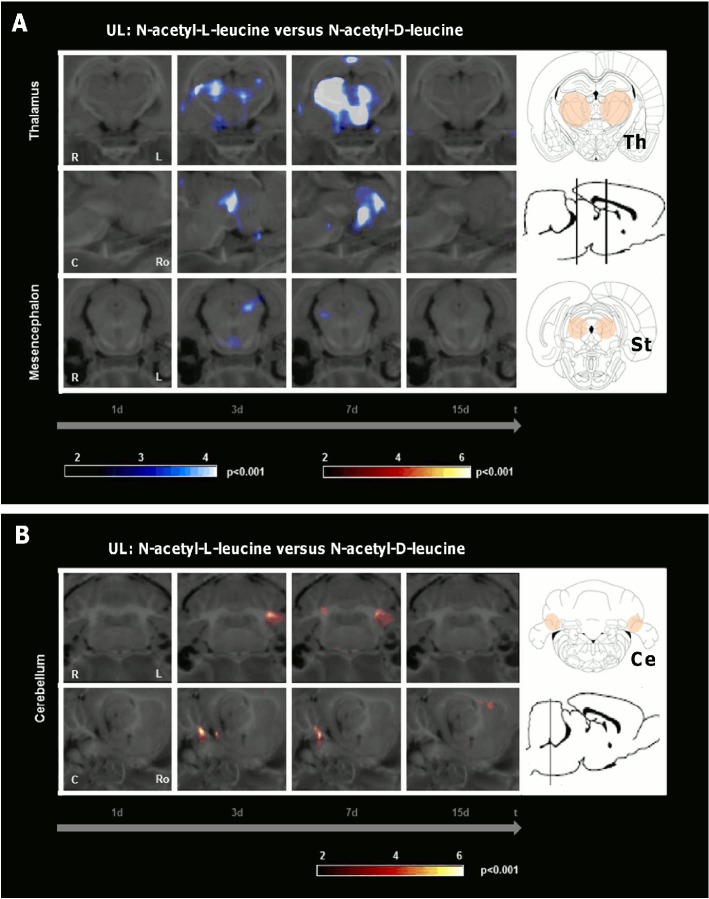
Regional cerebral glucose metabolism after N-acetyl-L-leucine as compared to N-acetyl-D-leucine treatment following unilateral labyrinthectomy. The images depict group-wise comparisons of the regional cerebral glucose metabolism (rCGM) on days 1, 3, 7 and 15 after unilateral labyrinthectomy (UL). A) Comparison of the N-acetyl-L-leucine group and the N-acetyl-D-leucine group (24 mg i.v. per rat each) revealed a significant decrease of rCGM in the contralesional>ipsilesional posterolateral thalamus on day 3 and 7. B) On day 3 rCGM was significantly increased in the ipsilesional, on day 7 in the ipsilesional>contralesional paraflocculus/flocculus (vestibulocerebellum) in the N-acetyl-L-leucine group. R right, L left, C caudal, Ro rostral, Ce cerebellum, St subthalamic region, Th thalamus; p-value < 0.001

Comparison of the UL-groups treated with N-acetyl-L-leucine and N-acetyl-D-leucine (24 mg i.v. per rat each) indicated the following differences in rCGM (Fig. 6, Table 1): First, in the L-group a significant decrease of rCGM appeared in the contralesional>ipsilesional posterolateral thalamus and subthalamic region on day 3. It was even more prominent in the contralesional>ipsilesional posterolateral thalamus on day 7. Second, in the L-group rCGM was significantly increased on day 3 in the ipsilesional, on day 7 in the ipsilesional>contralesional paraflocculus/flocculus (vestibulocerebellum). Third, there were no other differences of rCGM at any other time point.

### Effects of N-acetyl-L-leucine on cerebral plasticity after UL and sham UL

**Fig 7:**
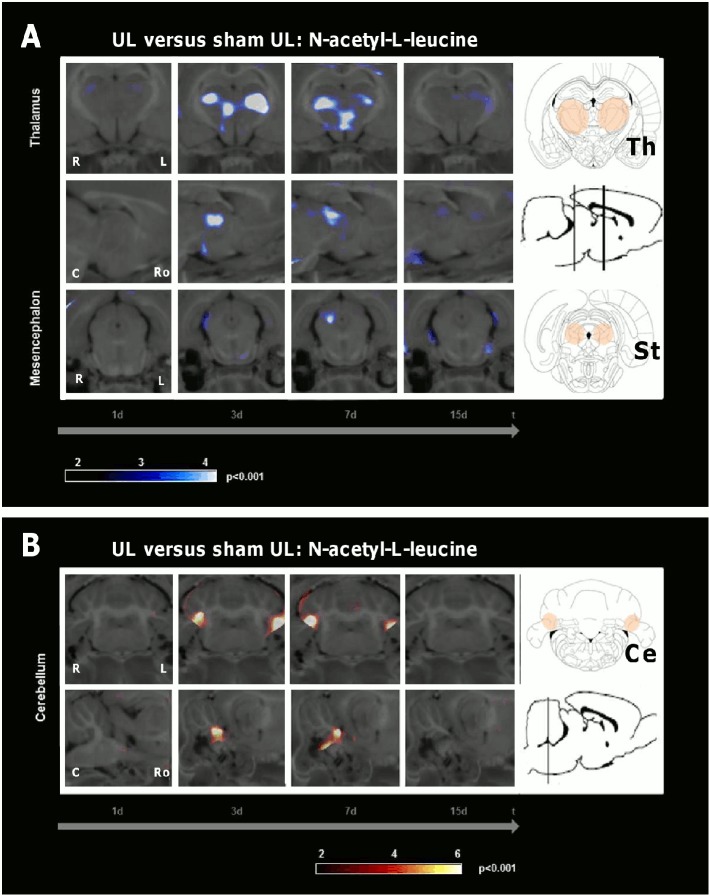
Comparison of the N-acetyl-L-leucine effect on regional cerebral glucose metabolism following unilateral labyrinthectomy and sham unilateral labyrinthectomy. The images depict the comparison of the regional cerebral glucose metabolism (rCGM) on days 1, 3, 7 and 15 between groups treated with N-acetyl-L-leucine (24 mg i.v. per rat) after unilateral labyrinthectomy (UL) and after sham UL (without inner ear damage). A) rCGM was significantly decreased in the posterolateral thalamus bilaterally on days 3 and 7 and the contralesional subthalamic region on day 3 in the UL-group as compared to the sham UL-group. B) On days 3 and 7 rCGM was significantly increased in the vestibulocerebellum bilaterally in the UL-group. R right, L left, C caudal, Ro rostral, Ce cerebellum, St subthalamic region, Th thalamus; p-value < 0.001

Comparison of rCGM in the UL-group and the sham UL-group (without inner ear damage) both treated with N-acetyl-L-leucine (24 mg i.v. per rat each) showed the following differences (Fig. 7, Table 1): In the UL-group rCGM relatively decreased in the thalamus and increased in the paraflocculus/flocculus bilaterally on days 3 and 7.

## Discussion

The major findings of this study were the following: 1) N-acetyl-DL-leucine accelerates the postural compensation after unilateral vestibular damage; 2) N-acetyl-L-leucine is the pharmacologically active enantiomer that induces this effect; 3) the potential mechanism of N-acetyl-L-leucine action for improving vestibular compensation consists of an activation of the vestibulocerebellum and a deactivation of the posterolateral thalamus; 4) N-acetyl-L-leucine effects thalamic and cerebellar metabolism only after peripheral vestibular but not auditory damage.

### Differential effects of N-acetyl-DL-leucine, N-acetyl-L-leucine and N-acetyl-D-leucine on vestibular compensation

Our data show that N-acetyl-DL-leucine at a dose of 24 mg i.v. per rat significantly accelerated compensation of postural symptoms but not of nystagmus or head roll tilt after UL. Application of this drug shortened the time course of postural compensation by approximately 40%. These data fully agree with findings of previous animal and human studies, which also showed a preferential effect of N-acetyl-DL-leucine on postural stability and mobility [16, 17, 20]. From a clinical point of view, postural rebalancing is a key aspect of functional restoration after an acute vestibular syndrome, since it increases the patient’s free mobility and thereby also allows further compensation.

Testing of the D- and L-enantiomer in our study revealed that N-acetyl-L-leucine is evidently the active component that accelerates postural compensation in a dose-dependent manner. An estimation shows that a dosage of 60mg/kg body weight N-acetyl-L-leucine i.v. in the rat likely is in the range of the dose response curve where the faciliatatory effect is saturated (data not shown). This explains why the effect of 60mg/kg body weight N-acetyl-L-leucine is not double the effect of 60mg/kg body weight N-acetyl-DL-leucine in our experiments. Despite interspecies differences in metabolism, the daily administration of 60 mg/kg N-acetyl-L-leucine in the rat is equal to about 8 g of N-acetyl-DL-leucine in patients. This is the dosage currently used in clinical practice. The L-leucine enantiomer specificity of the therapeutic effect indicates a stereospecific pharmacological mode of action (see below).

### Cerebral metabolic correlates of improved vestibular compensation after N-acetyl-L-leucine treatment

The behavioural improvement with N-acetyl-L-leucine treatment follows an increase of rCGM in the vestibulocerebellum and a decrease in the posterolateral thalamus on days 3 and 7. It is noteworthy that the timely dynamics of this pattern lags behind the application of the drug (days 1–3), but precedes the postural improvement (after day 7). Therefore, N-acetyl-L-leucine most likely augments plasticity mechanisms, which do not occur directly and immediately but probably via more complex and delayed molecular and cellular modulations, which improve postural compensation secondarily.

The importance of the vestibulocerebellum for compensation in the subacute phase after vestibular damage is well known. The cerebellum has intense anatomical projections to the VN, and it also substantially contributes to motor learning in relation to the vestibular system [21]. Ablation of the vestibulocerebellum or disruption of climbing fibre inputs in the first week after UL severely disturbed VC [22]. Further, transgenic mice with cerebellar dysfunction showed no further restoration of the vestibulo-ocular reflex 5 d post UL [23]. Patients with medullary vestibular lesions exhibit cerebellar activation during the late phase of compensation [24]. Sequential whole-brain [^18^F]-FDG-μPET scanning in the rat model of UL showed an increase of rCGM in the vestibulocerebellum on days 7 and 9 after UL [13]. A comparable vestibulocerebellar rCGM increase was already found in our N-acetyl-L-leucine group on days 3 and 5 after UL (Fig. 4). This effect was again specific to N-acetyl-L-leucine (Fig. 5). It did not appear after a sham UL without inner ear damage (Fig. 7). Therefore, acceleration of postural compensation by N-acetyl-L-leucine is paralleled by a forward displacement of vestibulocerebellar activation and takes place only in case of a peripheral vestibular deficit.

The posterolateral thalamus is a region, where vestibular, proprioceptive and visual information as well as cerebellar efferent signals converge [25]. Therefore, it is most likely involved in multisensory integration. An altered glucose metabolism occurs in the thalamus of patients after unilateral vestibular damage. This might reflect a mismatch between the vestibular and other sensory information [26]. Our data show a prominent decrease of thalamic rCGM on days 3 and 7 following treatment with N-acetyl-L-leucine (Fig. 4), but not following treatment with N-acetyl-D-leucine (Fig. 5) or after sham UL without inner ear damage (Fig. 7). The time course of the thalamic changes in the N-acetyl-L-leucine group was similar to that of cerebellar activation, possibly indicating that both effects are related. We can only speculate on the functional relevance of the thalamic metabolic changes after administration of N-acetyl-L-leucine. One hypothesis is that thalamic downregulation may reduce the transmission of the sensory mismatch information to the cortex, thereby reducing the vertigo/dizziness sensation and improving postural balance.

### Putative mechanisms of N-acetyl-L-leucine action on vestibular compensation

The authors are aware that rCGM depicts metabolic changes, but does not allow conclusions about the underlying mechanisms (e.g., changes in fire rate, synaptic input or structural plasticity). However, it has been shown that ‘pharmacological imaging’ can be used to determine whether a given drug has the ability to modulate the response in a known functional cerebral circuitry [27]. Serial μPET allows study of therapeutic interventions on VC in order to identify cerebral regions within vestibular networks in vivo on a whole-brain level, where direct or indirect pharmacological changes take place over time. This may be of special interest, if the cerebral mode of action is not exactly known—as in the case of N-acetyl-DL-leucine or N-acetyl-L-leucine.

So far, it was hypothesized that N-acetyl-DL-leucine restores the membrane potential of both hyperpolarized and depolarized vestibular neurons [18]. The effect may be mediated by modulation of the ion channel activity. However, such a pharmacological action should cause immediate or short-latency effects on nystagmus and postural symptoms; however, neither we nor others have observed this [16]. We furthermore did not find rCGM changes in the VN induced by N-acetyl-L-leucine treatment. This could of course be due to the minor therapeutic effect on VN rCGM or the limited spatial resolution. On the other hand, we showed previously that the rCGM is asymmetric in the VN at 4 h and becomes symmetric already 1 d after UL—a long time before postural compensation is established [13]. Restoring VN membrane property therefore may not be sufficient to explain the whole mechanisms of postural compensation and the mode of action of N-acetyl-L-leucine.

The close phylogenetic and anatomic similarities and interactions between the vestibular and vestibulocerebellar neurons suggest that N-acetyl-L-leucine might also have effects on cerebellar neurons. A recent study in patients with cerebellar ataxia showed that N-acetyl-DL-leucine improved symptoms [28]. N-acetyl-L-leucine chemically is derived from the essential amino acid leucine by addition of an acetyl group. Addition of acetyl or carbon groups or chains to small molecules can change their properties quite radically. A strong argument for the specificity of the mode of action is the fact, that only the L-enantiomer but not the D-enantiomer shows a therapeutic effect on postural compensation. In addition N-acetyl-L-leucine only changes the regional cerebral glucose metabolism after vestibular damage. These observations strongly indicate a binding of N-acetyl-L-leucine to a stereospecific site of a receptor or enzyme, which is upregulated during VC. In the vestibulocerebellum i.a. the following molecular changes have been reported in the acute phase after unilateral vestibular damage: 1) protein c kinase expression [29]; 2) cerebellar-specific glutamate receptor delta 2 [30]; 3) calbindin expression [31]. N-acetyl-L-leucine, which is a branched-chain amino acid, may modulate glutamate neurotransmission in the cerebellum via the branched-chain amino acid transferases [32–34]. Proteomic binding studies are necessary to further clarify the interations of N-acetyl-L-leucine in the brain. It is hypothezised that N-acetyl-L-leucine may interact with and modulate cellular mechanisms as named above. Functionally it thereby may induce changes in long-term potentiation or depression via changes in glutamatergic neurotransmission or cellular calcium levels in the cerebellum. Activation of metabotropic glutamate receptors (mGluR) is required for cerebellar plasticity during VC and can indirectly increase the intrinsic excitability of VN neurons [35–37]. The cerebellar rCGM increase in our study could be the correlate of an enhanced cerebellar excitability due to increased glutamate transmission and alteration of discharge rates of cerebellar neurons. Alternative actions of N-acetyl-L-leucine via binding to membrane phospholipids are conceivable. The decrease of rCGMglc in the thalamus could be due to a direct effect of N-acetyl-L-leucine. However, it is more likely that this effect is also a consequence of altered cerebellar-thalamic projections.

### Conclusion

This study shows that N-acetyl-L-leucine accelerates postural compensation after unilateral vestibular damage in a dose-dependent and specific manner and, thus, is the effective component of N-acetyl-DL-leucine. We propose that its mode of action operates via the vestibulocerebellum and thalamus, a suggestion that is for the first time based on sequential in vivo whole-brain μPET imaging. The exact electrophysiological and pharmacological mechanism remains to be elucidated.

## Supporting Information

S1 Dataset Supporting information. (TIF)

S2 Dataset Supporting information. (DOCX)
