# Supplementary material for: N-Acetyl-L-Leucine Accelerates Vestibular Compensation after Unilateral Labyrinthectomy by Action in the Cerebellum and Thalamus
Source: PLoS One. 2015 Mar 24;10(3):e0120891. doi: 10.1371/journal.pone.0120891 (PMC4372420; doi:10.1371/journal.pone.0120891)
Supplement: S2 Dataset — (DOCX) [file pone.0120891.s002.docx]

| **Table supplementary material** | | | | | | | | |
| --- | --- | --- | --- | --- | --- | --- | --- | --- |
|  | **UL: Sham treatment** | | | | **UL: N-acetyl-L-leucine** | | | |
|  | 1 day | 3 day | 7 day | 15 day | 1 day | 3 day | 7 day | 15 day |
| Inf. colliculus | *10,69 (R)* | *9,55 (R)* | *8,51 (R)* | *8,80 (R)* | *7,88 (R)* | *13,83 (R)* | *8,63 (R)* | *10,49 (R)* |
| Auditory cortex | *11,77 (R)* | *6,26 (R)* | *9,77 (R)* | *8,03 (R)* | *8,43 (R)* | *9,09 (R)* | *5,59 (R)* | *10,40 (R)* |
|  | | | | | | | | |
|  | **UL: N-acetyl-D-leucine** | | | | **Sham UL: N-acetyl-L-leucine** | | | |
|  | 1 day | 3 day | 7 day | 15 day | 1 day | 3 day | 7 day | 15 day |
| Inf. colliculus | *8,60 (R)* | *11,62 (R)* | *12,90 (R)* | *10,09 (R)* | *8,52 (R)* | *7,03 (R)* | *5,82 (R)* | *6,62 (R)* |
| Auditory cortex | *8,51 (R)* | *10,72 (R)* | *10,57 (R)* | *7,40 (R)* | *9,78 (R)* | *8,13 (R)* | *5,60 (R)* | *6,71 (R)* |
|  |  |  |  |  |  |  |  |  |
| The table shows the regional cerebral glucose metabolism (rCGM) changes in the inferior colliculus and auditory cortex for the different experimental groups as a function of time (compared to the baseline condition before unilateral labyrinthectomy (UL)/ sham UL). Top left: Sham treatment following UL; top right: N-acetyl-L-leucine treatment following UL; bottom left: N-acetyl-D-leucine treatment following UL; bottom right: N-acetyl-L-leucine following sham UL. Decrease of rCGM is depicted in Italic. The significance of the clusters is depicted by t-values at a p-value of 0.001. Degrees of freedom: (n_1_-1)+(n_2_-1) L left side, R right side | | | | | | | | |
